# Supplementary material for: Splice-Junction-Based Mapping of Alternative Isoforms in the Human Proteome
Source: Cell Rep. Author manuscript; Available in PMC 2020 Jan 15. (PMC6961840; doi:10.1016/j.celrep.2019.11.026)
Supplement: 2 [file NIHMS1546469-supplement-2.zip › DF1/PXD010154/Liver/Liver_47_AAAS_VYNASRPSSGCAQVLSHPGHTPVTSLAWAPSGGR.pdf]

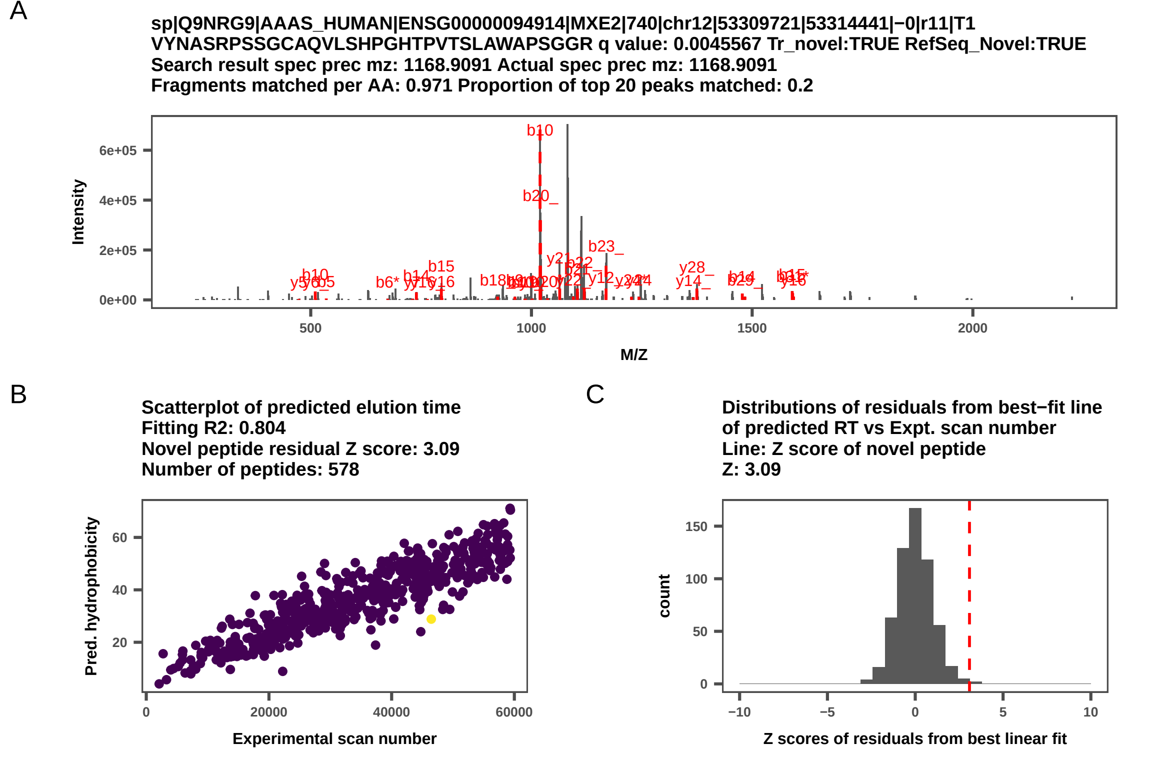
A
sp|Q9NRG9|AAAS_HUMAN|ENSG00000094914|MXE2|740|chr12|53309721|53314441|−0|r11|T1
VYNASRPSSGCAQVLSHPGHTPVTSLAWAPSGGR q value: 0.0045567 Tr_novel:TRUE RefSeq_Novel:TRUE
Search result spec prec mz: 1168.9091 Actual spec prec mz: 1168.9091
Fragments matched per AA: 0.971 Proportion of top 20 peaks matched: 0.2
Intensity
6e+05
4e+05
2e+05
0e+00
b10
b20_
b23_
b15
b6*
y28_
y14_
M/Z
500
1000
1500
2000
B
Scatterplot of predicted elution time
Fitting R2: 0.804
Novel peptide residual Z score: 3.09
Number of peptides: 578
Pred. hydrophobicity
60
40
20
0
20000
40000
60000
Experimental scan number
C
Distributions of residuals from best−fit line
of predicted RT vs Expt. scan number
Line: Z score of novel peptide
Z: 3.09
count
150
100
50
0
−10
−5
0
5
10
Z scores of residuals from best linear fit
